# Alternative approach to the buckling phenomenon by means of a second order incremental analysis

**DOI:** 10.1038/s41598-023-43243-2

**Published:** 2023-09-26

**Authors:** Faustino N. Gimena, Mikel Goñi, Pedro Gonzaga, José-Vicente Valdenebro

**Affiliations:** Department of Engineering, Campus Arrosadía, Public University of Navarre, C.P. 31006 Pamplona, Navarre Spain

**Keywords:** Mechanical engineering, Civil engineering

## Abstract

This article addresses the problem of determining the solicitation and deformation of beams with geometric imperfection, also called real beams under a compression action. This calculation is performed by applying the Finite Transfer Method numerical procedure under first-order effects with the entire compression action applied instantaneously and applying the action gradually under second-order effects. The results obtained by this procedure for real sinusoidal or parabolic beams are presented and compared. To verify the potential of the numerical procedure, the first and second-order effects of a beam with variable section are presented. New analytical formulations of the bending moment and the transverse deformation in the beam with sinusoidal imperfection subjected to compression are also obtained, under first and second-order analysis. The maximum failure load of the beams is determined based on their initial deformation. The results of solicitation and deformation of the real beam under compression are compared, applying the analytical expressions obtained and the numerical procedure cited. The beams under study are profiles with different geometric characteristics, which shows that it is possible to obtain maximum failure load results by varying the relationships between lengths, areas and slenderness. The increase in second-order bending moments causes the failure that originates in the beam, making it clear that this approach reproduces the buckling phenomenon. The article demonstrates that through the Finite Transfer Method the calculation of first and second-order effects can be addressed in beams of any type of directrix and of constant or variable section.

## Introduction

Elastic instability is the set of structural situations of geometric non-linearity that manifests itself in that the displacements in a resistant member are not proportional to the acting forces^1^. Buckling is a phenomenon of elastic instability that can occur in slender structural members subjected to compression^2–4^. Slenderness is a mechanical characteristic of structural beams that relates the cross-sectional stiffness of a beam to its overall length^5^.

In the buckling phenomenon, significant displacements are produced perpendicular to the direction of compression. This phenomenon appears mainly in pillars and columns. It translates into an additional moment in the pillar when it is subjected to the action of significant axial compression loads^6^.

In structural engineering, elastic instability, of both resistant compressed members and of the structures made up of them, is one of the most complex problems and one of the greatest practical importance. Naturally, the analysis and reflection on the unstable elastic behaviour of beams have attracted the attention of so many researchers over time^7–15^. Despite all the contributions after Euler, the approach to the buckling problem has not changed^16–19^.

As a starting premise, we can define an ideal beam as a resistant member with a straight directrix. To this end, it must be manufactured without initial stresses or heterogeneities, and without any geometric imperfection. When an ideal beam is subjected to simple compression, the displacement of each point of the directrix has only a longitudinal component. But in every real beam there are heterogeneities and initial manufacturing stresses, and its directrix is not perfectly rectilinear. In the real beam, the directrix has a deviation from the straight line at each point. The compression load generates normal force and bending. The displacements produced in this case have longitudinal and transverse components. This combination of effects is often called first-order solicitation. On the other hand, the combination of effects that adds the bending generated by the transverse displacements to the previous one is called second-order solicitation^2^. If the compression load increases linearly, the increase in first-order solicitation is also linear. The same does not happen with the solicitation of the second-order. If the bending generated by transverse displacements is considered, as the load increases, the effects increase more rapidly^20,21^.

When checking the compressed beam using first-order analysis, there is no elastic instability. In the second-order analysis, the bending effect produced by the transverse displacement during the application of the compression load is variable and increasing. This makes the superposition principle invalid. Therefore, an elastic instability is generated.

The imperfection of the directrix shape can be dealt with by assimilating the actual beam to a curved beam. By applying the first-order analysis on the curved beam, all kinds of effects are obtained, both compression and bending solicitations, as well as longitudinal and transverse displacements.

This article deals with the calculation of the real beam assimilated to the curved beam under second-order conditions. To do this, the load is divided into increments first. Each load increment is applied, and the solicitations and deformations are obtained. Then, the shape of the directrix is modified by adding the displacements obtained, and the next load increment is applied again. It is a successive process of iterations until exhausting the load increments to be applied. For this iterative calculation, a numerical procedure of boundary conditions has been used^22–26^.

By solving the differential equation of the elastica posed by Euler, a sinusoidal function^27^ is obtained, from which the critical load is deduced. Under this approach, by assimilating the calculation of a compressed beam to that of a curved beam with a sinusoidal directrix, under second-order conditions, it has been verified that there is no elastic instability.

An alternative approach is presented to address this same problem, which reinterprets the theory that is usually used in the study of elastic instability due to buckling.

Unlike Euler’s formulation, in which the deformation generates solicitation, we start from a differential equation of the elastica with sinusoidal strain. That is, the bending moment is generated solely by the initial imperfection. This is intended to introduce the real directrix of the beam into the behaviour model.

The loads are applied incrementally, choosing increments so small that we can assume a linear behaviour in each increase in load.

The deformed configuration obtained for each load increase is added to the starting geometry for the next calculation iteration.

The second-order analysis is applied by solving a succession of first-order analysis of a beam whose geometry changes with each load increment with respect to the previous ones.

This alternative approach is specified in a final analytical expression of the deformed directrix of the beam, under the effect of second-order, once the application of the entire load after the iterations is exhausted.

The results obtained by means of this analytical expression derived from the revision of Euler's approach are compared with those obtained numerically by calculating the real beam assimilated to the curved beam under second-order conditions.

With the aim of highlighting the research presented, as a practical case, the structural behaviour of a variable section beam is analysed.

## Analysis of the beam by means of the finite transfer method under second-order effects

**Figure 1:**
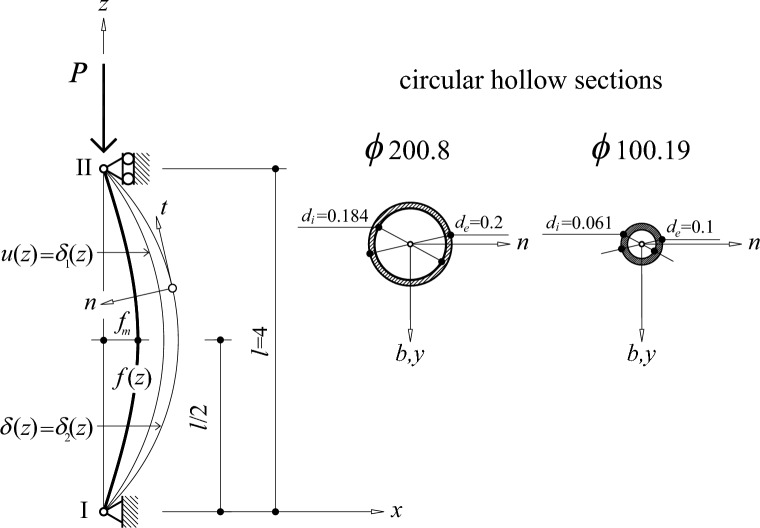
Bi-articulated real beam.

In this section the imperfect beam is analysed under a compression load (see Fig. 1), assimilating it to a curved beam. Together with the geometric imperfection of the directrix, it is considered that the beam has no initial manufacturing stresses, that its material is homogeneous and isotropic, and that the section is constant.

To carry out the structural calculation, the numerical procedure called Finite Transfer Method^28^ is used. This procedure solves a system of linear ordinary differential equations with boundary conditions and can address the wide casuistry of the structural problem of the beam^29–31^. Finite Transfer Method uses a repetition strategy on the discretised directrix that allows relating the solicitation and deformation values of the ends of the structural beam through an algebraic system. The dimension of said system is always constant and independent of the intervals obtained by the discretisation. It uses a fourth-order scheme to obtain a suitable numerical approximation.

To carry out the first-order analysis, a computer program that applies the Finite Transfer Method is used on the initial directrix of the beam to be calculated, with the entire compression load. To obtain second-order solicitation and deformation values, the load has been divided into equal parts (10,000 parts). Firstly, the numerical program has been executed with the first portion of load on the initial position of the directrix. With the results obtained, the new form of the directrix has been calculated and the numerical procedure has been executed on it for the second time. This process has been repeated until the application of the entire load has been completed. With this, a second-order analysis has been carried out on the real beam.

Two cases of steel beam types and different hollow circular section with the same area have been chosen. As shown in Fig. 1, these beams are supported by a hinge at the lower end I, and linear support at the upper end II.

Table 1 shows the characteristics in terms of shape and material of the two study type beams: hollow circular profile $\phi 200.8$ and $\phi 100.19$. In the structural analysis carried out, the shear coefficients are considered null.

**Table 1:** Formal characteristics and materials of the beams.

Beam	*l* (m)	*f*_*m*_ (mm)	Steel	*E* (kN/mm^2^)	*G* (kN/mm^2^)
	4	4	S355	210	81
Circular hollow sections	*d*_*e*_ (mm)	*d*_*i*_ (mm)	*A* (cm^2^)	*I*_*t*_ (cm^4^)	*I* = *I*_*n*_ = *I*_*b*_ (cm^4^)
ϕ200.8	200	184	48.25	4454.89	2227.44
ϕ100.19	100	62.10	48.25	835.77	417.89

The load that intervenes in the calculation represents the maximum load that these beams can support without imperfections (ideal beam) and without using safety factors to determine the resistance of the material. The value of this load is $P_{0} = f_{y} A = {1713}\,{\text{kN}}$.

To assimilate the beam with geometric imperfection to a curved beam, two cases are analysed: sinusoidal directrix and parabolic directrix. In both examples, the maximum initial strain in $f_{m}$ is 4 mm.

### Sinusoidal directrix beam

In this first analysis, the real beam is identified with a curved beam whose directrix has a sinusoidal shape whose equation is:

1 $$f\left( z \right) = f_{m} \sin \left( {{{\pi z} \mathord{\left/ {\vphantom {{\pi z} l}} \right. \kern-0pt} l}} \right).$$

Table 2 shows the solicitation and deformation values of the beam profile $\phi 200.8$ under both first-order and second-order effects. These solicitation and strain values are presented at eight points uniformly distributed on the directrix of the beam (0, 0.5, 1, 1.5, 2, 2.5, 3, 3.5 and 4 m).

**Table 2:** Sinusoidal beam profile $\phi 200.8$: first and second-order solicitations and deformations.

*z* (m)	Solicitations and strains											
	First-order						Second-order					
	*N* = *V*_*t*_ (kN)	*V*_*n*_ (kN)	*M*_*b*_ = *M*_*y*_ (kN.m)	*θ*_*y*_ (10^–3^ rad)	*δ*_*x*_ (mm)	*δ*_*z*_ (mm)	*N* = *V*_*t*_ (kN)	*V*_*n*_ (kN)	*M*_*b*_ = *M*_*y*_ (kN.m)	*θ*_*y*_ (10^–3^ rad)	*δ*_*x*_ (mm)	*δ*_*z*_ (mm)
0	− 1713.04	5.382	0.000	1.865	0.000	0.000	− 1713.02	9.744	0.000	2.547	0.000	0.000
0.5	− 1713.04	4.972	− 2.622	1.723	0.906	− 0.848	− 1713.02	9.002	− 3.580	2.353	1.237	− 0.851
1	− 1713.04	3.805	− 4.845	1.319	1.674	− 1.695	− 1713.03	6.890	− 6.616	1.801	2.286	− 1.700
1.5	− 1713.05	2.059	− 6.331	0.714	2.188	− 2.541	− 1713.04	3.729	− 8.644	0.975	2.987	− 2.547
**2**	− 1713.05	0.000	** − 6.852**	0.000	**2.368**	− 3.387	− 1713.05	0.000	** − 9.356**	0.000	**3.233**	− 3.392
2.5	− 1713.05	− 2.059	− 6.331	− 0.714	2.188	− 4.232	− 1713.04	− 3.729	− 8.644	− 0.975	2.987	− 4.238
3	− 1713.04	− 3.805	− 4.845	− 1.319	1.674	− 5.078	− 1713.03	− 6.890	− 6.616	− 1.801	2.286	− 5.085
3.5	− 1713.04	− 4.972	− 2.622	− 1.723	0.906	− 5.926	− 1713.02	− 9.002	− 3.580	− 2.353	1.237	− 5.934
4	− 1713.04	− 5.382	0.000	− 1.865	0.000	− 6.774	− 1713.02	− 9.744	0.000	− 2.547	0.000	− 6.784

Significant values are in bold.

The solicitation is made up of the normal $N = V_{t}$ and shear forces $V_{n}$, and the bending moment $M_{b} = M_{y}$ in the intrinsic axes. The deformation is composed of the gyration $\theta_{y}$, and the transverse $\delta_{x}$ and longitudinal $\delta_{z}$ displacements, under the general reference system.

Analysing the results, the values of normal stress and shear stress are not comparable. Therefore, it is considered sufficiently approximate to determine the normal stress and ignore the tangential stress.

Comparing in the centre the effects of second-order with those of first-order, an increase of 36.54% is observed in the bending moment and in the transverse displacement. Regarding the longitudinal displacement, the increase is only 0.16%.

Table 3 shows the solicitation and deformation of the sinusoidal beam with a hollow circular profile $\phi 100.19$.

**Table 3:** Sinusoidal beam profile $\phi 100.19$: first and second-order solicitations and strains.

*z* (m)	Solicitations and strains											
	First-order						Second-order					
	*N* = *V*_*t*_ (kN)	*V*_*n*_ (kN)	*M*_*b*_ = *M*_*y*_ (kN.m)	*θ*_*y*_ (10^–3^ rad)	*δ*_*x*_ (mm)	*δ*_*z*_ (mm)	*N* = *V*_*t*_ (kN)	*V*_*n*_ (kN)	*M*_*b*_ = *M*_*y*_ (kN.m)	*θ*_*y*_ (10^–3^ rad)	*δ*_*x*_ (mm)	*δ*_*z*_ (mm)
0	− 1713.04	5.382	0.000	9.939	0.000	0.000	− 1708.33	127.055	0.000	71.119	0.000	0.000
0.5	− 1713.04	4.972	− 2.622	9.182	4.840	− 0.859	− 1709.02	117.400	− 18.762	65.706	34.603	− 2.151
1	− 1713.04	3.805	− 4.845	7.028	8.944	− 1.714	− 1710.69	89.884	− 34.669	50.290	63.945	− 3.939
1.5	− 1713.05	2.059	− 6.331	3.804	11.685	− 2.564	− 1712.36	48.661	− 45.300	27.217	83.558	− 5.214
**2**	− 1713.05	0.000	** − 6.852**	0.000	**12.648**	− 3.409	− 1713.05	0.000	** − 49.033**	0.000	**90.447**	− 6.127
2.5	− 1713.05	− 2.059	− 6.331	− 3.804	11.685	− 4.254	− 1712.36	− 48.661	− 45.300	− 27.217	83.558	− 7.040
3	− 1713.04	− 3.805	− 4.845	− 7.028	8.944	− 5.104	− 1710.69	− 89.884	− 34.669	− 50.290	63.945	− 8.316
3.5	− 1713.04	− 4.972	− 2.622	− 9.182	4.840	− 5.959	− 1709.02	− 117.400	− 18.762	− 65.706	34.603	− 10.104
4	− 1713.04	− 5.382	0.000	− 9.939	0.000	− 6.818	− 1708.33	− 127.055	0.000	− 71.119	0.000	− 12.255

Significant values are in bold.

Logically, the solicitation values under first-order effects of the two curved beams with sinusoidal directrix are the same.

In the second order analysis the relationship between the maximum shear force and the maximum normal force is 7.42%. In relation to the bending moment and the transverse displacement in the centre, an increase of 615% is observed between the second and first-order values. Regarding the longitudinal displacement, the increase is only 80%.

Under second-order effects, the relationship between the bending moment in the centre of the beams with profile $\phi 200.8$ and $\phi 100.19$ is 534%. The relationship between transverse displacements in the centre of the beams type $\phi 200.8$ and $\phi 100.19$ is 2797%.

The relationship between the first-order transverse displacement in the centre and the length of the real beam is 0.32%. Under second order analysis, this relationship is 2.26%.

### Parabolic directrix beam

In this section, the calculation of the imperfect beam is identified with that of a curved beam with a parabolic directrix whose equation is:

2 $$f\left( z \right) = \frac{{4f_{m} }}{{l^{2} }}\left( {l - z} \right)z.$$

Table 4 shows, in a similar way to the values presented in Table 2, the solicitation and deformation values are shown under effects of both first-order and second-order in the beam profile $\phi 200.8$.

**Table 4:** Parabolic beam profile $\phi 200.8$: first and second-order solicitations and deformations.

*z* (m)	Solicitations and strains											
	First-order						Second-order					
	*N* = *V*_*t*_ (kN)	*V*_*n*_ (kN)	*M*_*b*_ = *M*_*y*_ (kN.m)	*θ*_*y*_ (10^–3^ rad)	*δ*_*x*_ (mm)	*δ*_*z*_ (mm)	*N* = *V*_*t*_ (kN)	*V*_*n*_ (kN)	*M*_*b*_ = *M*_*y*_ (kN.m)	*θ*_*y*_ (10^–3^ rad)	*δ*_*x*_ (mm)	*δ*_*z*_ (mm)
0	− 1713.03	6.852	0.000	1.953	0.000	0.000	− 1713.02	9.933	0.000	2.657	0.000	0.000
0.5	− 1713.04	5.139	− 2.998	1.785	0.945	− 0.849	− 1713.02	8.844	− 3.995	2.435	1.287	− 0.851
1	− 1713.04	3.426	− 5.139	1.343	1.734	− 1.696	− 1713.03	7.044	− 6.972	1.840	2.366	− 1.701
1.5	− 1713.05	1.713	− 6.424	0.717	2.254	− 2.542	− 1713.05	0.131	− 8.808	0.986	3.079	− 2.547
**2**	− 1713.05	0.000	** − 6.852**	0.000	**2.435**	− 3.387	− 1713.05	0.000	** − 9.428**	0.000	**3.327**	− 3.393
2.5	− 1713.05	− 1.713	− 6.424	− 0.717	2.254	− 4.233	− 1713.05	− 0.131	− 8.808	− 0.986	3.079	− 4.238
3	− 1713.04	− 3.426	− 5.139	− 1.343	1.734	− 5.079	− 1713.03	− 7.044	− 6.972	− 1.840	2.366	− 5.085
3.5	− 1713.04	− 5.139	− 2.998	− 1.785	0.945	− 5.926	− 1713.02	− 8.844	− 3.995	− 2.435	1.287	− 5.934
4	− 1713.03	− 6.852	0.000	− 1.953	0.000	− 6.774	− 1713.02	− 9.933	0.000	− 2.657	0.000	− 6.786

Significant values are in bold.

Comparing the second-order effects with the first-order ones in the centre, an increase of 37.59% is observed in relation to the bending moment and 36.66% in relation to the transverse displacement. In the longitudinal displacement the increase is only 0.17%.

Table 5 shows the solicitation and strain in the parabolic beam with a hollow circular profile $\phi 100 \cdot 19$.

**Table 5:** Parabolic beam profile $\phi 100.19$: first and second-order solicitations and strains.

*z* (m)	Solicitations and strains											
	First-order						Second-order					
	*N* = *V*_*t*_ (kN)	*V*_*n*_ (kN)	*M*_*b*_ = *M*_*y*_ (kN.m)	*θ*_*y*_ (10^–3^ rad)	*δ*_*x*_ (mm)	*δ*_*z*_ (mm)	*N* = *V*_*t*_ (kN)	*V*_*n*_ (kN)	*M*_*b*_ = *M*_*y*_ (kN.m)	*θ*_*y*_ (10^–3^ rad)	*δ*_*x*_ (mm)	*δ*_*z*_ (mm)
0	− 1713.03	6.852	0.000	10.408	0.000	0.000	− 1707.90	132.712	0.000	73.573	0.000	0.000
0.5	− 1713.04	5.139	− 2.998	9.514	5.049	− 0.862	− 1708.75	121.239	− 19.706	67.856	35.771	− 2.253
1	− 1713.04	3.426	− 5.139	7.156	9.265	− 1.717	− 1710.57	92.061	− 35.952	51.784	66.030	− 4.096
1.5	− 1713.05	1.713	− 6.424	3.822	12.038	− 2.566	− 1712.33	49.591	− 46.620	27.963	86.204	− 5.391
**2**	− 1713.05	0.000	** − 6.852**	0.000	**13.003**	− 3.411	− 1713.05	0.000	** − 50.335**	0.000	**93.278**	− 6.306
2.5	− 1713.05	− 1.713	− 6.424	− 3.822	12.038	− 4.256	− 1712.33	− 49.591	− 46.620	− 27.963	86.204	− 7.222
3	− 1713.04	− 3.426	− 5.139	− 7.156	9.265	− 5.105	− 1710.57	− 92.061	− 35.952	− 51.784	66.030	− 8.517
3.5	− 1713.04	− 5.139	− 2.998	− 9.514	5.049	− 5.960	− 1708.75	− 121.239	− 19.706	− 67.856	35.771	− 10.360
4	− 1713.03	− 6.852	0.000	− 10.408	0.000	− 6.822	− 1707.90	− 132.712	0.000	− 73.573	0.000	− 12.613

Significant values are in bold.

Under second-order effects, the relationship between the maximum shear force and the maximum normal force is 7.66%. In relation to the bending moment in the centre, an increase of 635% is observed between second and first-order values. The relationship between the bending moment in the centre of the beams with profile $\phi 200.8$ and $\phi 100.19$ is 534%.

In the centre there is a 617% increase in second-order transverse displacement compared to first order. Regarding the longitudinal displacement, the increase is only 85%. The relationship between transverse displacements in the centre of the beams type $\phi 200.8$ and $\phi 100.19$ is 2803% under the second-order analysis.

The relationship between the first-order transverse displacement in the centre and the length of the real beam is 0.33%. This relationship under the second-order analysis is 2.33%.

### Results comparison

**Figure 2:**
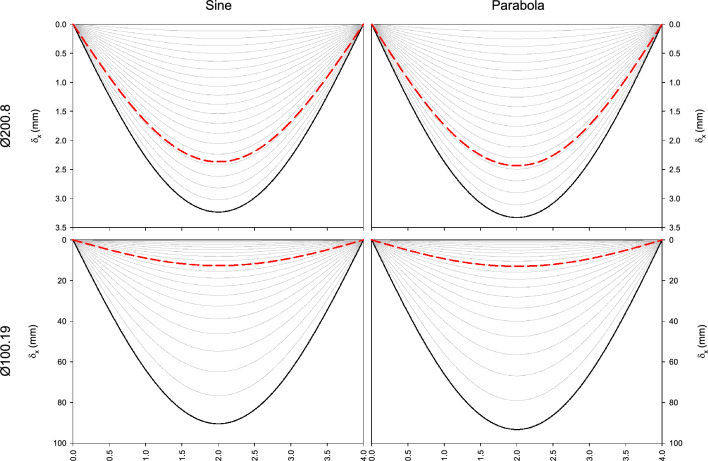
Transverse strains of sinusoidal and parabolic beams.

Figure 2 shows the values of transverse strain of the beams with sinusoidal and parabolic directrix under the compression load, whose numerical values are written in Tables 2, 3, 4 and 5. The bending moment graphs have not been presented because this is proportional to the transverse displacement.

Both first-order effects (red dashed line) and second-order effects (solid line) have been represented. The second-order transverse displacement functions associated with different proportions of load have also been graphed. The percentage of second-order effects is greater when the load portion is greater.

Figure 2 shows that under second-order effects, greater strain is experienced than under first-order effects. The beams with the lowest moment of inertia suffer greater deformation. The differences between assimilating the imperfect beam to a beam with a sinusoidal or parabolic directrix are negligible. In the second order analysis of the beam profile $\phi 200.8$, the difference between bending moments is 0.77% and between transverse displacements is 2.66%. In the beam profile $\phi 100.19$, these differences slightly increase, being these percentages 2.91% in the case of calculating bending moments and 3.13% in the case of transverse displacements.

### Sinusoidal directrix beam with variable section

In this section the calculation of the real beam is identified with that of a curved piece with a sinusoidal directrix Eq. (1). The variable circular section beam is studied. This section varies linearly along the directrix, from the initial end $\phi 100$ to the final end $\phi 78$.

Table 6 shows the stress and deformation values under both first-order and second-order effects.

**Table 6:** Variable section sinusoidal beam: first and second-order solicitations and deformations.

*z* (m)	Solicitations and deformations											
	First-order						Second-order					
	*N* = *V*_*t*_ (kN)	*V*_*n*_ (kN)	*M*_*b*_ = *M*_*y*_ (kN.m)	*θ*_*y*_ (10^–3^ rad)	*δ*_*x*_ (mm)	*δ*_*z*_ (mm)	*N* = *V*_*t*_ (kN)	*V*_*n*_ (kN)	*M*_*b*_ = *M*_*y*_ (kN.m)	*θ*_*y*_ (10^–3^ rad)	*δ*_*x*_ (mm)	*δ*_*z*_ (mm)
0	− 1713.04	5.382	0.000	12.487	0.000	0.000	− 1671.30	375.906	0.000	218.190	0.000	0.000
0.5	− 1713.04	4.972	− 2.622	11.794	6.128	− 0.553	− 1675.46	356.869	− 42.331	207.048	106.383	− 12.319
1	− 1713.04	3.805	− 4.845	9.616	11.542	− 1.131	− 1687.29	295.942	− 79.967	171.481	201.444	− 22.283
1.5	− 1713.05	2.059	− 6.331	5.972	15.495	− 1.735	− 1702.41	190.612	− 107.881	110.346	272.660	− 28.184
**2**	− 1713.05	0.000	** − 6.852**	1.145	17.314	− 2.370	− 1712.45	45.353	− 121.296	26.488	307.640	− 30.216
2.5	− 1713.05	− 2.059	− 6.331	− 4.295	16.537	− 3.044	− 1708.49	− 124.845	− 116.628	− 71.775	296.671	− 31.223
3	− 1713.04	− 3.805	− 4.845	− 9.510	13.057	− 3.767	− 1687.82	− 292.882	− 92.641	− 169.671	236.101	− 35.935
3.5	− 1713.04	− 4.972	− 2.622	− 13.461	7.241	− 4.547	− 1660.18	− 422.284	− 51.589	− 246.286	131.596	− 48.151
4	− 1713.04	− 5.382	0.000	− 15.020	0.000	− 5.386	− 1646.25	− 473.694	0.000	− 277.154	0.000	− 67.039

Significant values are in bold.

The solicitation values under first-order effects of the three curved beams with sinusoidal directrix are equal.

Comparing the second-order effects with the first-order effects in the span, an increase of 1670.19% is observed in relation to the bending moment and 1676.82% in relation to the transversal displacement.

**Figure 3:**
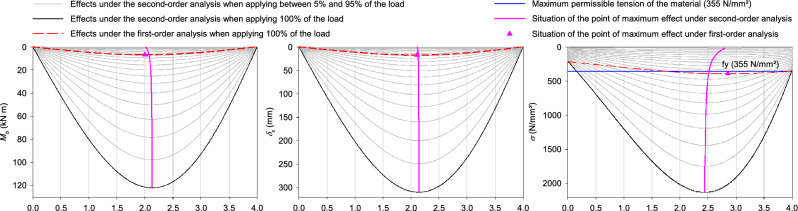
Bending moments, transversal deformations and stresses in the sinusoidal beam with variable section.

Figure 3 shows that since the beam has a variable section, symmetry is not maintained with respect to the span of the solicitations, deformations and stresses.

Under second-order effects the maximum values are: for the bending moment 122 kN at 2.13 m from the initial end, for the tangential displacement 309 mm at 2.14 m from the initial end.

The maximum normal stress of failure or collapse of the beam occurs with 52% of the applied load and 2.52 m from the initial end.

## Analysis of the beam through an analytical procedure

Due to imperfection, the directrix of the beam has a deviation from the straight line. According to Euler’s approach, an ideal beam subjected to a compression load can be in stable or unstable equilibrium, elastically.

**Figure 4:**
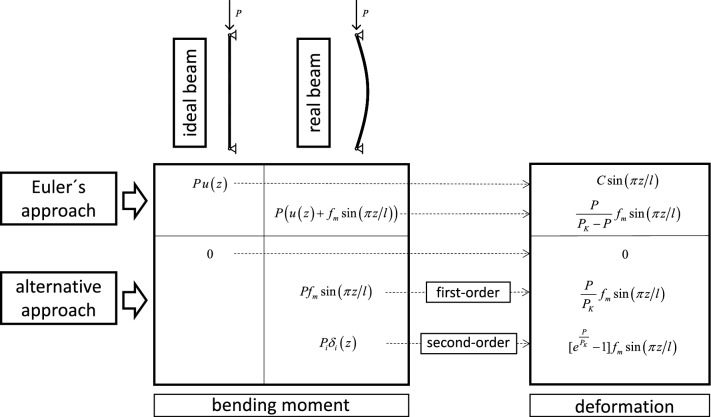
Structural approaches on the ideal and real beam.

Figure 4 shows the starting expressions of the bending moments, under different approaches for the ideal beam and for the real beam. The expressions of the tangential displacements that are obtained by Euler's approach and by the analytical procedure that is proposed are also shown.

### Beam under Euler’s approach

It is usual to derive the critical Euler load from the bi-hinged beam compressed by a point load $P$. The directrix has only transverse strain $u\left( z \right)$. It is considered that the section of the beam is constant and made of the same material. No other type of load takes place. The weight of the beam itself is considered negligible.

The differential expression of the deformed directrix can be written as:

3 $$\frac{{d^{2} u\left( z \right)}}{{dz^{2} }} = - \frac{P}{EI}u\left( z \right).$$

The analytical solution of transverse displacement is obtained by solving this differential equation. Its expression can be noted as^32^:

4 $$u\left( z \right) = C_{1} \sin \left( {\sqrt {{P \mathord{\left/ {\vphantom {P {EI}}} \right. \kern-0pt} {EI}}} z} \right) + C_{2} \cos \left( {\sqrt {{P \mathord{\left/ {\vphantom {P {EI}}} \right. \kern-0pt} {EI}}} z} \right),$$

where $C_{1} ,\;C_{2}$ are the two constants of integration.

From the application of the support conditions $u\left( 0 \right) = 0;\;u\left( l \right) = 0;\;$ it can be derived that $C_{2} = 0$ and that $\sin \left( {\sqrt {{P \mathord{\left/ {\vphantom {P {EI}}} \right. \kern-0pt} {EI}}} l} \right) = 0$. If it were not so, the directrix would be a straight line and there would be no transverse deformation. For this last support condition to be met, the value that the load must acquire is $P_{K} = {{\pi^{2} EI} \mathord{\left/ {\vphantom {{\pi^{2} EI} {l^{2} }}} \right. \kern-0pt} {l^{2} }}$. This value is the Euler critical load.

In the real bi-articulated beam, the effect of the imperfections is considered equivalent, with sufficient approximation, to the one produced if its directrix instead of being initially straight is a sinusoid. The analytical notation of the directrix is expressed in Eq. (1). Under Euler’s approach and in the real beam, the differential expression of the deformed directrix is^6^:

5 $$\frac{{d^{2} \delta \left( z \right)}}{{dz^{2} }} = - \frac{P}{EI}\left[ {f_{m} \sin \left( {{{\pi z} \mathord{\left/ {\vphantom {{\pi z} l}} \right. \kern-0pt} l}} \right) + u\left( z \right)} \right].$$

The analytical solution of this differential equation, applying the support condition of the real bi-hinged beam is:

6 $$\delta \left( z \right) = \frac{P}{{P_{K} - P}}f_{m} \sin \left( {{{\pi z} \mathord{\left/ {\vphantom {{\pi z} l}} \right. \kern-0pt} l}} \right).$$

This function represents the transverse displacement suffered by the real beam in compression. When the load $P$ is equal to the critical load $P_{K}$, a discontinuity in the function is produced, and therefore the real beam is in a state of elastic instability.

The bending moment $M\left( z \right)$ produced by the load at each point of the directrix of the beam is:

7 $$M\left( z \right) = \frac{{PP_{K} }}{{P_{K} - P}}f_{m} \sin \left( {{{\pi z} \mathord{\left/ {\vphantom {{\pi z} l}} \right. \kern-0pt} l}} \right).$$

The bending moment remains finite while *P*<*P*_K_, and becomes infinite, however small is *f*_m_, when *P *= *P*_K_. Therefore *P*_K_ is the upper limit of the load that can be applied to a double-hinged beam. In the theoretical case of an ideal beam with *f*_m_ = 1, there is a stable equilibrium if *P*<*P*_K_, but it becomes unstable with *P *= *P*_K_.

### Beam under second-order effects

Remembering that an ideal beam is a resistant member with a straight directrix manufactured without initial stresses or heterogeneities, and without any geometric imperfection, when it is subjected to a compression load, the displacement of each point of the directrix has only a longitudinal component. In this case, there is neither transverse displacement nor bending moment. The maximum load that the ideal beam can withstand is obtained by multiplying the resistance of the material by the area of the section.

As previously commented, in the real beam the effect of the imperfections is equivalent to considering its directrix sinusoidal instead of straight. Under this approach, the differential expression of the deformed directrix can be noted as:

8 $$\frac{{d^{2} \delta_{1} \left( z \right)}}{{dz^{2} }} = - \frac{P}{EI}f\left( z \right) = - \frac{P}{EI}f_{m} \sin \left( {{{\pi z} \mathord{\left/ {\vphantom {{\pi z} l}} \right. \kern-0pt} l}} \right).$$

The solution of this equation for the beam with biarticulated support is:

9 $$\delta_{1} \left( z \right) = \frac{P}{{P_{K} }}f_{m} \sin \left( {{{\pi z} \mathord{\left/ {\vphantom {{\pi z} l}} \right. \kern-0pt} l}} \right),$$

where *P*_K_ is the Euler critical load expressed in Eq. (5).

In this case, the entire load *P* has been applied to the bi-hinged beam instantaneously. This way the first order strain $\delta_{1} \left( z \right)$ is obtained.

To determine the second order deformation $\delta \left( z \right) = \delta_{2} \left( z \right)$, the load *P* must be applied gradually. To do this, it is divided into *n* equal parts, and the calculation is carried out applying its first increase. From this calculation, the deformation produced is deduced and a new directrix is generated. By repeating this calculation *i* times, the deformation is:

10 $$\delta_{i} \left( z \right) = \left[ {\left( {1 + \frac{P}{{nP_{K} }}} \right)^{i} - 1} \right]f_{m} \sin \left( {{{\pi z} \mathord{\left/ {\vphantom {{\pi z} l}} \right. \kern-0pt} l}} \right).$$

By gradually applying the entire load, taking this division to the limit, the second order deformation and expressed as:

11 $$\delta \left( z \right) = [e^{{\frac{P}{{P_{K} }}}} - 1]f_{m} \sin \left( {{{\pi z} \mathord{\left/ {\vphantom {{\pi z} l}} \right. \kern-0pt} l}} \right).$$

The first-order deformation expressions Eq. (9) and second-order Eq. (11) are continuous functions.

In first-order deformation, if the value of the force action is Euler's critical load, the displacement obtained coincides with the initial sinusoidal directrix of the beam. In second-order deformation, if the action is the critical load, the directrix of the beam is e–1 ≃ 1.718 times its initial position.

The second-order bending moment can be noted as:

12 $$M\left( z \right) = - EI\frac{{d^{2} \delta \left( z \right)}}{{dz^{2} }} = P_{K} [e^{{\frac{P}{{P_{K} }}}} - 1]f_{m} \sin \left( {{{\pi z} \mathord{\left/ {\vphantom {{\pi z} l}} \right. \kern-0pt} l}} \right).$$

Like what is detected in the deformation equation under second-order analysis, there is no discontinuity in the bending moment formulation.

The maximum normal stress of the beam occurs in the centre section and at the furthest point from the barycentre. Its value is:

13 $$\sigma = \frac{P}{A}\left[ {1 + \frac{{P_{K} }}{P}[e^{{\frac{P}{{P_{K} }}}} - 1]\frac{{Af_{m} }}{W}} \right].$$

Being $W$ the resistant module.

To exhaust a beam at its maximum stress, the function that relates the initial imperfection in the centre $f_{m}$ and the compression load $P$ can be noted as:

14 $$f_{m} = \frac{{f_{y} A - P}}{{P_{K} [e^{{\frac{P}{{P_{K} }}}} - 1]}}\frac{W}{A}.$$

As it happens in the first order strain expressions Eq. (9) and second order Eq. (11), in Eq. (14) there is no discontinuity.

## Results and relationships between second-order effects

As previously noted, the differences in both first-order and second-order effects between the sinusoidal and parabolic directrix beam are negligible. It has also been verified that the first-order effects are not close to the reality of the structural problem posed. From this point on, the formulations associated with the sinusoidal directrix beam will be used, presenting only second-order effects.

Table 7 compares the results obtained using the Finite Transfer Method numerical procedure with those obtained by applying the analytical formulas associated with Eqs. (11) and (12).

**Table 7:** Sinusoidal beams of profiles $\phi 200.8$ and $\phi 100.19$: second-order effects.

*M*_*b*_ = *M*_*y*_ (kN.m)	*f*200.8		*f*100.19		*δ*_*x*_ (mm)	*f*200.8		*f*100.19	
*z* (m)	Sinusoidal numerical	Sinusoidal analytical	Sinusoidal numerical	Sinusoidal analytical	*z* (m)	Sinusoidal numerical	Sinusoidal analytical	Sinusoidal numerical	Sinusoidal analytical
0	0.000	0.000	0.000	0.000	0	0.000	0.000	0.000	0.000
0.5	− 3.580	− 3.581	− 18.762	− 18.792	0.5	1.237	1.241	34.603	34.714
1	− 6.616	− 6.616	− 34.669	− 34.722	1	2.286	2.293	63.945	64.143
1.5	− 8.644	− 8.644	− 45.300	− 45.367	1.5	2.987	2.996	83.558	83.807
2	− 9.356	− 9.356	− 49.033	− 49.105	2	3.233	3.243	90.447	90.712
2.5	− 8.644	− 8.644	− 45.300	− 45.367	2.5	2.987	2.996	83.558	83.807
3	− 6.616	− 6.616	− 34.669	− 34.722	3	2.286	2.293	63.945	64.143
3.5	− 3.580	− 3.581	− 18.762	− 18.792	3.5	1.237	1.241	34.603	34.714
4	0.000	0.000	0.000	0.000	4	0.000	0.000	0.000	0.000

Any difference between the values obtained by the two procedures is less than 0.30%. There are practically no differences between the values of bending moments and transverse displacements obtained by numerical or analytical methods. When using the numerical procedure for the calculation, in addition to bending moments and transverse displacements, the other values of the effect are obtained: normal stresses, shear stresses, gyrations and longitudinal displacements. As previously noted, the effects that produce tangential stresses are negligible.

From the analysis of the results expressed in Table 7, it can be deduced that, at the level of structural verification, it is considered sufficiently approximate to determine the values of the second-order effect by the analytical procedure that has been developed.

Table 8 shows the values of the maximum compression load that can be supported by different profiles of the same length and area.

**Table 8:** Maximum compression load for different profiles of equal area.

*l* = 4 m		Section					
		ϕ200.8	ϕ175.9	ϕ158.10	ϕ125.14	ϕ100.19	ϕ78
*P*_K_ (kN) →		2885	2153	1713	981	541	240
*P*_0_ (kN) →		1713	1713	1713	1713	1713	1713
*f*_m_	*l*/1000	1537	1494	1450	1288	1018	603
	*l*/500	1401	1337	1276	1083	822	477
	*l*/250	1198	1118	1047	850	625	359
	*l*/100	853	771	706	547	391	222

The beams analysed are the profile types studied in Table 1. ($\phi 200.8$ and $\phi 100.19$), to which hollow circular profiles $\phi 175.9$, $\phi 158.10$, $\phi 125.14$ are added, and the circular solid profile $\phi 78$, for a length of 4 m. These beams are analysed under different initial imperfections $f_{m}$: ideal beam without deformation, and beams whose deflection-span ratio is 1/1000, 1/500, 1/250 and 1/100.

It is observed that the maximum load for the beams without initial imperfection is the same: $P_{0} = {1713}\,{\text{kN}}$. The same does not happen with the critical load $P_{K}$, since all the analysed profiles have different moments of inertia. In the cases studied, it is seen that the maximum load depends on both the initial imperfections and the section of the beams. As the outer diameter of the beam decreases, the maximum compression load decreases. When the initial imperfection of the beam increases, this maximum load supported by the profile also decreases.

Table 9 shows the values of the maximum compression load that can be supported by steel profiles taken from commercial standard series.

**Table 9:** Maximum compression load for different standard profiles.

*l* = 4 m		Section				
		ϕ200.8	ϕ200.6	ϕ200.5	ϕ100.6	ϕ100.5
*P*_K_ (kN) →		2885	2231	1887	255	219
*P*_0_ (kN) →		1713	1298	1087	629	530
*f*_m_	*l*/1000	1537	1168	979	428	364
	*l*/500	1401	1066	895	353	301
	*l*/250	1198	914	768	274	234
	*l*/100	853	653	550	175	150

Beams of the same length (4 m) and with the profiles $\phi 200.8$, $\phi 200.6$, $\phi 200.5$, $\phi 100.6$ and $\phi 100.5$ have been chosen for this analysis. These beams are also analysed under the same initial imperfections as in the previous study.

The maximum load that these beams support without initial imperfection is the product of the resistance of the material $f_{y} = {355}\,{{\text{N}} \mathord{\left/ {\vphantom {{\text{N}} {{\text{mm}}^{2} }}} \right. \kern-0pt} {{\text{mm}}^{2} }}$ by the area of the profile used. The resulting critical loads $P_{K}$ are different because the moments of inertia of the profiles are different. Similarly, to the case studies presented in Table 8, in these examples the maximum load also depends on both the initial imperfections and the sections of the profiles. If the area of the profile decreases and the initial imperfection of the beam increases, the maximum load decreases.

Table 10 shows the values of the maximum load that the same profile can support under different lengths.

**Table 10:** Maximum compression load for the same profile and different lengths.

ϕ200.8		*l* (m)			
		3	4	5	6
*P*_K_ (kN) →		5130	2885	1847	1282
*P*_0_ (kN) →		1713	1713	1713	1713
*f*_m_	*l*/1000	1592	1537	1470	1388
	*l*/500	1489	1401	1303	1198
	*l*/250	1321	1198	1078	965
	*l*/100	997	853	735	638

The standard profile $\phi 200.8$ has been chosen for this analysis, with lengths of 3 m, 4 m, 5 m and 6 m. The initial imperfections considered are the same as in Tables 7 and 8.

In these cases, when using the same profile, the maximum load for ideal beams or beams without initial imperfection is the same $P_{0} = {1713}\,{\text{kN}}$. It is observed that the critical loads $P_{K}$ are different because the lengths of the beams are different. If the length of the beam increases and the initial imperfection increases, the maximum load decreases.

Table 11 shows the values of the maximum load that can be supported by different profiles with the same area and the same slenderness.

**Table 11:** Maximum compression load for different profiles of equal area and slenderness.

*P*_K_ = 541kN, *λ* = 136		Section					
		ϕ200.8	ϕ175.9	ϕ158.10	ϕ125.14	ϕ100.19	ϕ78
*l* (m) →		9.235	7.977	7.116	5.384	4	2.664
*P*_0_ (kN) →		1713	1713	1713	1713	1713	1713
*f*_m_	*l*/1000	1057	1054	1051	1039	1018	973
	*l*/500	863	859	856	843	822	775
	*l*/250	665	662	658	646	625	581
	*l*/100	424	421	418	408	391	354

The beams analysed are the profiles studied in Table 8. The slenderness chosen is associated with the 4 m long beam and profile $\phi 100.19$, whose value is $\lambda = {l \mathord{\left/ {\vphantom {l {\sqrt {{I \mathord{\left/ {\vphantom {I A}} \right. \kern-0pt} A}} }}} \right. \kern-0pt} {\sqrt {{I \mathord{\left/ {\vphantom {I A}} \right. \kern-0pt} A}} }} = 136$. With this value the length of each beam is determined. Again, the initial imperfections $f_{m}$ are without deformation, and with a deflection-span ratio of 1/1000, 1/500, 1/250 and 1/100.

Also, in these cases, when using profiles with the same area, the maximum load for beams without initial imperfection is $P_{0} = {1713}\,{\text{kN}}$. The critical load $P_{K} = 541{\text{kN}}$ is maintained in the beams studied. If the initial imperfection increases, the maximum compression load decreases.

Table 12 shows the values of the maximum load that profile beams with the same length and the same slenderness can support.

**Table 12:** Maximum compression load for different profiles of equal slenderness and length.

*l* = 4 m, *λ* = 136		Section					
		ϕ110.34	ϕ105.26	ϕ100.19	ϕ95.13	ϕ90.7	ϕ85.2
*P*_K_ (kN) →		911	722	541	370	207	52
*P*_0_ (kN) →		2884	2285	1713	1169	654	166
*f*_m_	*l*/1000	1670	1340	1018	705	399	103
	*l*/500	1338	1077	822	571	325	84
	*l*/250	1009	816	625	437	250	65
	*l*/100	621	506	391	275	158	41

In the cases studied the chosen slenderness is associated with the beam profile $\phi 100.19$. The analysed beams have the following hollow circular profiles: $\phi 110.34$, $\phi 105.26$, $\phi 100.19$, $\phi 95.13$, $\phi 90.7$ and $\phi 85.2$. These beams are analysed under the same initial imperfections $f_{m}$ as in the previous cases.

It is observed that both the critical loads and the maximum loads are different in all the study cases. Whether the initial imperfection increases or the outer diameter of the profile decreases, the maximum compression load decreases.

## Conclusions

From the traditional study of the real beam with initial sinusoidal imperfection, the analytical formulations of the transverse deformation Eq. (11) and the bending moment Eq. (12) have been deduced. These expressions are associated with second-order effects and represent continuous functions at all points. Therefore, from this continuity condition, it is deduced that the collapse of the structure does not occur under any specific critical load. This collapse is due to the failure that originates in the beam due to the increase in second-order bending moments.

Under this alternative approach to the analysis of the buckling phenomenon, it is clear that to accurately determine the bending moment under the second-order analysis it is necessary to know or establish an initial deformation of the beam. For each material, the maximum failure load of the beam under compression depends on the initial imperfection, and other properties such as the length and geometric characteristics of the section.

To determine the maximum failure load or solicitation and deformation values under second-order effects, in beams and/or pieces with any initial imperfection or any type of section, constant or variable, the numerical procedure Finite Transfer Method can be applied using the repetition strategy developed in this work.

## Data Availability

The authors declared that all data generated or analysed during this study are included in this published article.
